# Preparation of TiO_2_/Carbon Nanotubes/Reduced Graphene Oxide Composites with Enhanced Photocatalytic Activity for the Degradation of Rhodamine B

**DOI:** 10.3390/nano8060431

**Published:** 2018-06-13

**Authors:** Yanzhen Huang, Dongping Chen, Xinling Hu, Yingjiang Qian, Dongxu Li

**Affiliations:** 1 College of Materials Science and Engineering, Huaqiao University, Xiamen 361021, China; 1611302044@hqu.edu.cn; 2 Fujian Key Laboratory of Photoelectric Functional Materials, Huaqiao University, Xiamen 361021, China; 1511302044@hqu.edu.cn (D.C.); 1611302043@hqu.edu.cn (X.H.); 17013081050@hqu.edu.cn (Y.Q.)

**Keywords:** titanium dioxide, carbon nanotubes, graphene, photocatalyst

## Abstract

In this report, ternary titanium dioxide (TiO_2_)/carbon nanotubes (CNTs)/reduced graphene oxide (rGO) composites were fabricated by a facile and environmentally friendly one-pot solvethermal method for the removal of Rhodamine B (RhB). Its structures were represented by X-ray powder diffraction (XRD), Raman spectrometry, scanning electron microscopy (SEM) and transmission electron microscopy (TEM). The photocatalytic performance was tested by the degradation efficiency of RhB under UV-vis light irradiation. The experimental results indicated that photocatalytic activity improved as the ratio of CNTs:TiO_2_ ranged from 0.5% to 3% but reduced when the content increased to 5% and 10%, and the TiO_2_/CNTs/rGO-3% composites showed superior photocatalytic activity compared with the binary ones (i.e., TiO_2_/CNTs, TiO_2_/rGO) and pristine TiO_2_. The rate constant *k* of the pseudo first-order reaction was about 1.5 times that of TiO_2_. The improved photocatalytic activity can be attributed to the addition of rGO and CNTs, which reduced the recombination of photo-induced electron-hole pairs, and the fact that CNTs and rGO, with a high specific surface area and high adsorption ability to efficiently adsorb O_2_, H_2_O and organics, can increase the hydroxyl content of the photocatalyst surface.

## 1. Introduction

Photocatalysts have aroused extensive interest for their effective treatment of organic contaminations which were degraded into small molecules of carbon dioxide (CO_2_) and water [1,2]. Anatase titanium dioxide (TiO_2_), with a 3.2 eV band gap, has been extensively researched as a photocatalyst because of its high photocatalytic activity, high stability, low toxicity and low cost [3,4,5]. However, as we all know, electron-hole recombination and the low availability of sunlight have been disadvantages that have reduced the photocatalytic activity [6,7]. Plentiful research works, such as those on noble metal deposition [8,9], transition metal or non-metallic elements dopants [10,11], metal oxide deposition [12,13] and preparation of carbon-based TiO_2_ compounds [14,15,16], have been done to solve the aforementioned problems.

Graphene has received wide study due to its distinct properties such as its outstanding charge-carrier mobility (~250,000 cm^2^·V^−1^·s^−1^ at room temperature), high thermal conductivity (~5000 W·m^−1^·K^−1^), high mechanical stiffness (~1 TPa) and high specific surface area (~2600 m^2^·g^−1^) for the preparation of photocatalyst composites [17,18]. Therefore, graphene can be applied to synthesize photocatalysts to increase the electron-hole separation efficiency and enhance the photocatalytic activity. Singh et al. [19] reported the photodegradation rate of water-soluble graphene nanosheets (wsGNS) isolated from toxic black pollutants was almost 11 times that of insoluble graphene nanosheets (GNS) to methyl blue (MB) under visible light. Zhang et al. [20] applied TiO_2_/graphene composites to degrade methyl orange (MO) and found that the photocatalytic activity of TiO_2_/graphene was higher than that of P25 and graphene. Pan et al. [7] incorporated graphene into TiO_2_ nanowires and obtained a preferable property for the removal of MB compared to TiO_2_ nanowires. Shiraishi et al. [21] reported that TiO_2_/reduced graphene oxide compounds yielded cyclohexanone with twice the amount formed on bare TiO_2_. Owing to the high surface to volume ratio, nanoscale ZnO particles have become rather vital to synthesize compounds [22]. Malekshoar et al. [23] proved a 30% improvement on the degradation rate of phenol by ZnO-graphene compared with ZnO. Fu [24] prepared a ZnO/TiO_2_ coupled film and researched the photocatalytic degradation activity of RhB. Carbon quantum dots, a kind of zero-dimensional nanomaterials, have received much attention due to their optical, physical and chemical properties. Tyagi et al. [25] detected the catalytic performance of TiO_2_-water soluble carbon quantum dots (wsCQDs) was ~1.5 times more than that of TiO_2_. Gogoi et al. [26] applied polymer-supported carbon dots to produce hydrogen peroxide. Another latent carbon material, carbon nanotubes (CNTs), are a kind of one-dimensional nanomaterial with high surface area and excellent conductivity [27]. Xiong et al. [28] proved that Fe/N-CNTs demonstrated better photocatalytic activity compared with Fe/CNTs to CO conversion. Jiang et al. [29] demonstrated that, when the doped graphitic-like N content was 6.22 at.%, nitrogen-doped carbon nanotube (NCNT)-supported NiO (NiO/NCNTs) showed the best photocatalytic oxidation property to toluene. Zhang et al. [30] disclosed that Pt/N-multiwalled CNTs (MWCNTs) possessed a higher selective oxidation of glycerol compared with Pt/MWCNTs. Liu et al. [31] developed a one-pot chemical method to synthesize anatase TiO_2_ onto MWCNTs which showed higher activity for the photocatalytic degradation of MO compared to pristine TiO_2_. Tetty et al. [32] demonstrated the reaction rate of MWCNTs/TiO_2_ fabricated through layer-by-layer assembly was one time higher than that of TiO_2_ to degrade Procion Red MX-50 (PR). Yen et al. [33] prepared a MWCNTs/TiO_2_ hybrid which displayed better photocatalytic activity for nitric oxide (NO) oxidation. With the development of carbon materials, many scientists have applied themselves to unite CNTs/rGO with metal oxide to receive ternary composites with an out-bound property. The CNTs loaded on rGO sheets would serve as charge transfer channels, which might strengthen the electrical property of rGO. Moreover, CNTs could prevent the stacking of rGO sheets and provide a larger surface area which would be beneficial to the photocatalytic performance.

Herein, TiO_2_ have been deposited on CNTs/rGO by a simple one-pot method. The effect that the mass ratio of CNTs to TiO_2_ has on photocatalytic properties was explored, and an optimum ratio was obtained. Furthermore, the as-prepared TiO_2_/CNTs/rGO-3% composites showed superior photocatalytic activity compared with the binary ones (i.e., TiO_2_/CNTs, TiO_2_/rGO) and pristine TiO_2_.

## 2. Experimental Part

### 2.1. Sample Preparation

Graphene oxide (GO, with diameter of 10~25 μm, thickness of 0.8~1.2 nm) was purchased from Shanghai Jiuai Biotechnology Co., Ltd. (Shanghai, China). CNTs (with diameter 60~100 nm, length > 5 μm) were purchased from Shenzhen Nanotech Port Co., Ltd. (Shenzhen, China).

**Figure 1:**
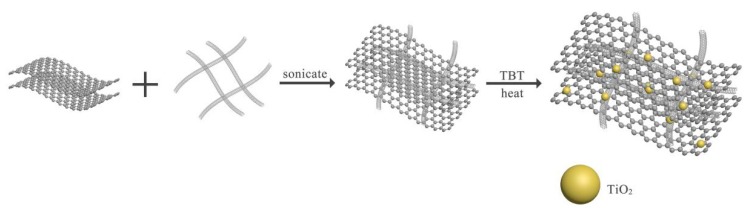
The schematic illustration of TiO_2_/carbon nanotubes (CNTs)/ reduced graphene oxide (rGO) composites.

TiO_2_/CNTs/rGO composites were synthesized by the solvethermal method. The CNTs were dissolved in 75 mL nitric acid (65%~68%), sonicated for 30 min and placed in a flask which was placed in a thermostatic water bath at 75 °C for 5~8 h. Then, the material was filtered, rinsed and dried at 80 °C overnight. The amount of 30 mg GO and a certain number of oxide-treated CNTs were dispersed in 25 mL of isopropyl alcohol and treated with an ultrasonic processor for 2 h. Then, tetrabutyl titanate (TBT, C_16_H_36_O_4_Ti) was mixed into the above suspension and stirred. This was followed by adding 1 mL distilled water by dropping. The mixture was transferred to the kettle and heated at 180 °C. The product was filtered, washed with distilled water until PH 7 was reached and dried at 60 °C overnight under vacuum. The quality ratio of CNTs:TiO_2_ was 0.5%, 1%, 2%, 3%, 5% and 10%, which were marked as TiO_2_/CNTs/rGO-*x*% (*x* = 0.5, 1, 2, 3, 5, 10). The schematic illustration of the TiO_2_/CNTs/rGO compound is shown in Figure 1. GO and oxide-treated CNTs were combined through π-π interaction by ultrasonification in homogeneous solution. The SEM images of GO, oxide-treated CNTs, CNTs/GO, TiO_2_/CNTs/rGO-3% were displayed in Figure S1. Wang et al. [6] also proved that the photocatalytic performance of TiO_2_/CNTs/graphene can be altered by diverse weight ratio of CNTs:TiO_2_. For further comparison, the pristine TiO_2_, TiO_2_/CNTs and TiO_2_/rGO were synthesized in accordance with the preparation process of TiO_2_/CNTs/rGO-3%.

### 2.2. Characterization

The crystalline phases were characterized by X-ray diffraction (XRD, Rigaku Miniflex 600, Rigaku, Tokyo, Japan) with Cu Kα radiation. Raman spectra were investigated by a micro-Raman spectroscopy system (Raman, inViainVi, London, UK). The surface morphology was obtained by field emission scanning electron microscopy (FESEM, Hitachi, Tokyo, Japan) and transmission electron microscopy (TEM, TECANI F30, FEI, Hillsboro, Nasdaq, USA). Degradation efficiency of RhB was acquired by UV-vis spectrophotometer (Shimadzu UV-2450, Tokyo, Japan).

### 2.3. Photocatalytic Activity

The experiments of removal of RhB for all photocatalyst composites were conducted under UV-vis light irradiation by a 300 W Xenon lamp (PLS-SXE300UV, Beijing, China) to research the photocatalytic activity. 10 mg of composites were added into a 60 mL 10 mg·L^−1^ RhB solution, sonicated for 10 min and then stirred in the dark for 30 min to guarantee the establishment of an adsorption/desorption equilibrium. During the reaction, 4 mL of solution was taken out every 10 min, centrifuged to remove catalyst particles and finally the degradation efficiency was analyzed with UV-vis spectrophotometer.

## 3. Results and Discussion

**Figure 2:**
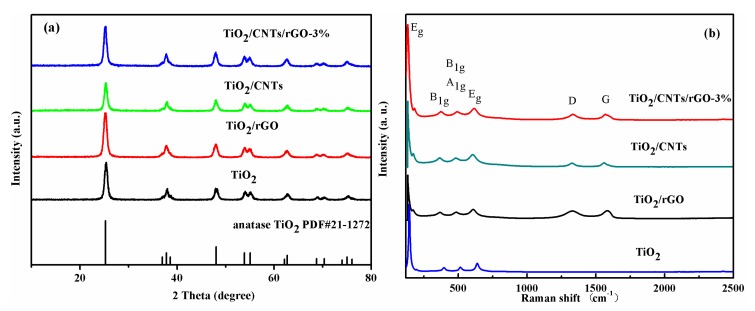
X-ray powder diffraction (**a**) (XRD) patterns; (**b**) Raman spectra of TiO_2_, TiO_2_/rGO, TiO_2_/CNTs and TiO_2_/CNTs/rGO-3%.

Figure 2a shows the XRD images of TiO_2_, TiO_2_/rGO, TiO_2_/CNTs and TiO_2_/CNTs/rGO-3%. The XRD patterns of other composites were shown in Figure S2a. It can be summarized precisely that all diffraction peaks are similar to those of anatase TiO_2_ (JCPDF No. 21-1272) [6]. The 2θ peaks appeared at 25.3°, 37.8°, 48.1°, 54.0°, 55.1°, 62.5°, 68.7°, 70.2°, and 75.2° corresponding to (101), (004), (200), (105), (211), (204), (116), (220), and (215) of TiO_2_. The fact that no diffraction peak of GO was observed demonstrated that GO might be reduced to rGO (in Figure S3) in the solvethermal process. In addition, the diffraction peak of rGO or CNTs at ~26° was possibly stacked with the leading peak of TiO_2_, which was reported in other literature [6,7].

Raman spectroscopy was carried out to analyze the crystal structure of TiO_2_ in the composites shown in Figure 2b. The Raman spectra of other composites are presented in Figure S2b. The Raman patterns of TiO_2_/rGO, TiO_2_/CNTs, TiO_2_/CNTs/rGO-3% showed the characteristic peaks of carbon materials with the presence of D band at 1355 cm^−1^ and G band at 1587 cm^−1^ which were ascribed to sp^3^ defects and the in-plane vibration of sp^2^ carbon atoms, respectively. The typical peaks at 144 (E_g_), 401 (B_1g_), 520 (B_1g_ + A_1g_) and 639 cm^−1^ (E_g_) of anatase TiO_2_ were consistent with the XRD patterns, further inferring the co-existence of TiO_2_, rGO and CNTs. The proportions of peak intensities of D and G for TiO_2_/rGO, TiO_2_/CNTs, TiO_2_/CNTs/rGO-3% have been calculated to be 0.98, 0.87 and 1.01, respectively. It is obvious that the *I*_D_/*I*_G_ ratio of TiO_2_/CNTs/rGO-3% composites was highest, which indicated that TiO_2_/CNTs/rGO-3% might be a characteristic of higher level disorder structures and more active sites [34] to improve photocatalytic properties.

**Figure 3:**
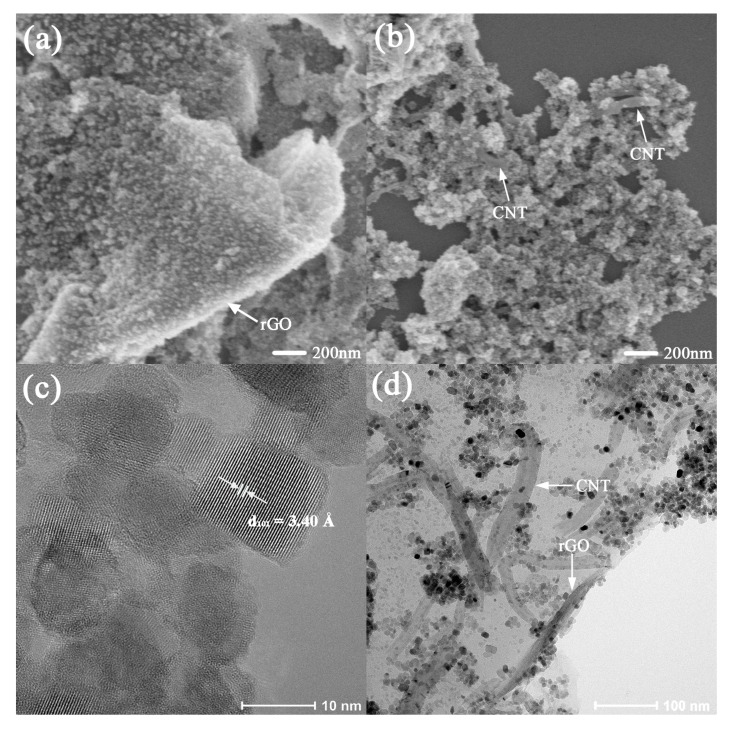
(**a**) Scanning electron microscopy (SEM) image of TiO_2_/rGO; (**b**) SEM image of TiO_2_/CNTs; (**c**) High resolution- transmission electron microscopy (HR-TEM) of TiO_2_; (**d**) transmission electron microscopy (TEM) image of TiO_2_/CNTs/rGO-3%.

To further demonstrate the surface topography, the SEM images of TiO_2_/rGO and TiO_2_/CNTs are shown in Figure 3a,b. TiO_2_ nanoparticles were uniformly loaded on two-dimensional rGO sheets with wrinkles which offered a larger surface area, contributing to electron transfer [35], as shown in Figure 3a. We can see that the CNTs substrates were covered by abundant TiO_2_ and there were bare CNTs. To further analyze the dispersion state of the ternary composites and particle size of TiO_2_, TEM was employed as shown in Figure 3c,d. The high-resolution transmission electron microscopy (HR-TEM) of anatase TiO_2_ demonstrated the lattice distance marked in the image was 0.34 nm, corresponding to the XRD results. What is more, the size of TiO_2_ was about 10 nm. TiO_2_ nanoparticles were not only loaded on rGO sheets but also absorbed on the surface of CNTs, as shown in Figure 3d, and CNTs were intercalated into rGO sheets, which could prevent the stacking of rGO, speed electron migration and improve photocatalytic performance [36].

**Figure 4:**
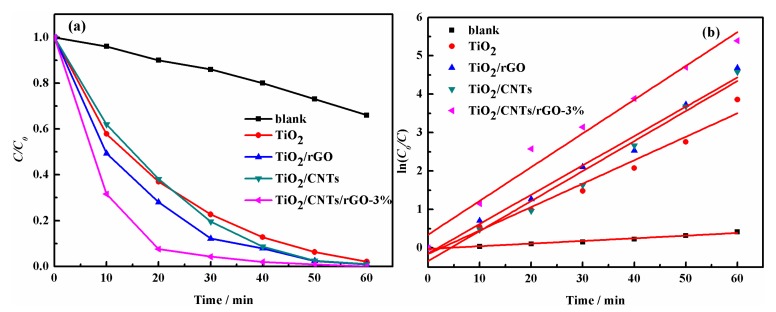
Blank test, TiO_2_, TiO_2_/rGO, TiO_2_/CNTs and TiO_2_/CNTs/rGO-3%: (**a**) plot of *C*/*C_0_* vs. irradiation time of RhB degradation; (**b**) linear transform ln(*C*_0_/*C*) = *kt* of the kinetic curves of RhB degradation.

The photoactivity TiO_2_/CNTs/rGO-*x*% (*x* = 0.5, 1, 2, 3, 5, 10) was evaluated by degradation of RhB (Figure S4), which showed time profiles of *C/C*_0_ under UV-vis light irradiation, where *C* is the concentration of RhB at irradiation time *t* and *C*_0_ the concentration at adsorption/desorption equilibrium before irradiation. The results indicated that photocatalytic activity improved as the ratio of CNTs:TiO_2_ ranged from 0.5% to 3%, but it reduced when the content increased, because the carrier transfer and separation were connected with suitable mass ratio and band gap [24]. In order to analyze TiO_2_/CNTs/rGO-3% composites in depth, the performance of blank test (RhB photocatalysis alone), TiO_2_, TiO_2_/rGO and TiO_2_/CNTs are represented in Figure 4a. We can clearly see that the activity order from high to low is TiO_2_/CNTs/rGO-3% > TiO_2_/rGO > TiO_2_/CNTs > TiO_2_ > blank test. As shown in Figure 4b, the rate constants *k* were 0.00689, 0.06099, 0.07631, 0.07809 and 0.08785 min^−1^ for blank test, TiO_2_, TiO_2_/CNTs, TiO_2_/rGO, and TiO_2_/CNTs/rGO-3%, respectively. After 30 min under UV-vis light, the RhB solution became colorless for TiO_2_/CNTs/rGO-3% (Figure S5). TiO_2_/CNTs/rGO-3% composites exhibited the best photoactivity whose *k* was almost 1.5 times that of TiO_2_. There are three reasons for this: rGO and CNTs with large surface areas and strong adsorption ability can adsorb O_2_, H_2_O and organics to increase the content of OH· and improve photocatalytic activity [37,38]; CNTs improved the oxidation-reduction ability and decreased the recombination efficiency of photo-inducd electron-hole pairs [39]; and CNTs prevented the stacking of rGO and enlarged the distance of rGO sheets that can enrich the active sites for photocatalytic reaction [40].

Herein, the photocatalytic degradation mechanism of TiO_2_/CNTs/rGO composites was speculated. Under UV-vis irradiation, TiO_2_ nanoparticles absorb light to produce photo-induced electron-hole pairs (Equation (1)) and RhB absorbs the photo flux (Equation (2)) [17]. Photo-generated electrons were seized by rGO and CNTs and transferred to the dye surface, thereby increasing the number of participating activated electrons (Equations (3) and (4)) [17]. Moreover, O_2_, H_2_O and organics can be adsorbed [37,38] by rGO and CNTs (Equations (5) and (6)) with a large surface area and excellent adsorption ability to further form H_2_O_2_ (Equation (5)) [17,39]. H_2_O_2_ is captured by electrons and holes promoting the formation of OH^−^, OH· (Equations (7–9)) [17]. The separated holes could react with OH^−^ and H_2_O to form OH· (Equations (10) and (11)) [6]. The activated oxidative species (·O_2_^−^, OH·) react with the excited dye molecules which finally results in its colorless appearance. The reaction mechanism could be supposed to be

(1) $$TiO_{2}\overset{h\upsilon }{\to }TiO_{2}\left(h^{+}+\;e^{-}\right)$$

(2) $$RhB\overset{h\upsilon }{\to }RhB^{*}\left(h^{+}+\;e^{-}\right)$$

rGO + e^−^ → rGO(e^−^) (3)

CNTs + e^−^ → CNTs(e^−^) (4)

rGO(e^−^) + O_2_ + 2H^+^ → rGO + H_2_O_2_ (5)

(6) $$H_{2}O_{2}\overset{hv}{\to }2{OH}^{-}$$

H_2_O_2_ + e^−^ → OH^−^ + OH· (7)

OH^−^ + h^+^ → OH· (8)

CNTs(e^−^) + O_2_ → CNTs + ·O_2_^−^ (9)

TiO_2_(h^+^) + OH^−^ → TiO_2_ + OH· (10)

TiO_2_(h^+^) + H_2_O → TiO_2_ + OH· (11)

## 4. Conclusions

In this study, the ternary TiO_2_/CNTs/rGO composites were fabricated by a facile and environmentally friendly one-pot solvethermal method. The TiO_2_/CNTs/rGO-3% composites showed the most outstanding photocatalytic activity. The improved photocatalytic activity was ascribed to the synergistic effect of TiO_2_, CNTs and rGO, in which TiO_2_ nanoparticles absorb light to produce photo-induced electron-hole pairs, whereas rGO and CNTs can reduce electron-hole recombination, adsorb O_2_, H_2_O and organics from the solution and air and enhance the photocatalytic performance. This study provided new ideas regarding the carbon-based TiO_2_ composites that are applied to the photocatalytic materials.

## Supplementary Materials

The following are available online at http://www.mdpi.com/2079-4991/8/6/431/s1, Figure S1: The SEM images of GO, oxide-treated CNTs, CNTs/GO, TiO_2_/CNTs/rGO-3%. Figure S2: The XRD patterns of rGO and GO. Figure S3: (a) The XRD patterns of TiO_2_/CNTs/rGO-*x*% (*x* = 0.5, 1, 2, 3, 5, 10); (b) The Raman spectra of TiO_2_/CNTs/rGO-*x*% (*x* = 0.5, 1, 2, 3, 5, 10). Figure S4: Plot of *C*/*C*_0_ vs irradiation time of RhB degradation for TiO_2_/CNTs/rGO-*x*% (*x* = 0.5, 1, 2, 3, 5, 10). Figure S5: The color change of RhB solution from 0, 10, 20, 30, 40, 50, 60 min under UV-vis light (from left to right).
