# Supplementary material for: Preparation of TiO2/Carbon Nanotubes/Reduced Graphene Oxide Composites with Enhanced Photocatalytic Activity for the Degradation of Rhodamine B
Source: Nanomaterials (Basel). 2018 Jun 13;8(6):431. doi: 10.3390/nano8060431 (PMC6027058; doi:10.3390/nano8060431)
Supplement: Supplementary file 1 [file nanomaterials-08-00431-s001.pdf]

Supplementary Materials: Preparation of TiO_2/Carbon Nanotubes/Reduced Graphene Oxide Composites with Enhanced Photocatalytic Activity for the Removal of Rhodamine B

Yanzhen Huang, Dongping Chen, Xinling Hu, Yingjiang Qian and Dongxu Li

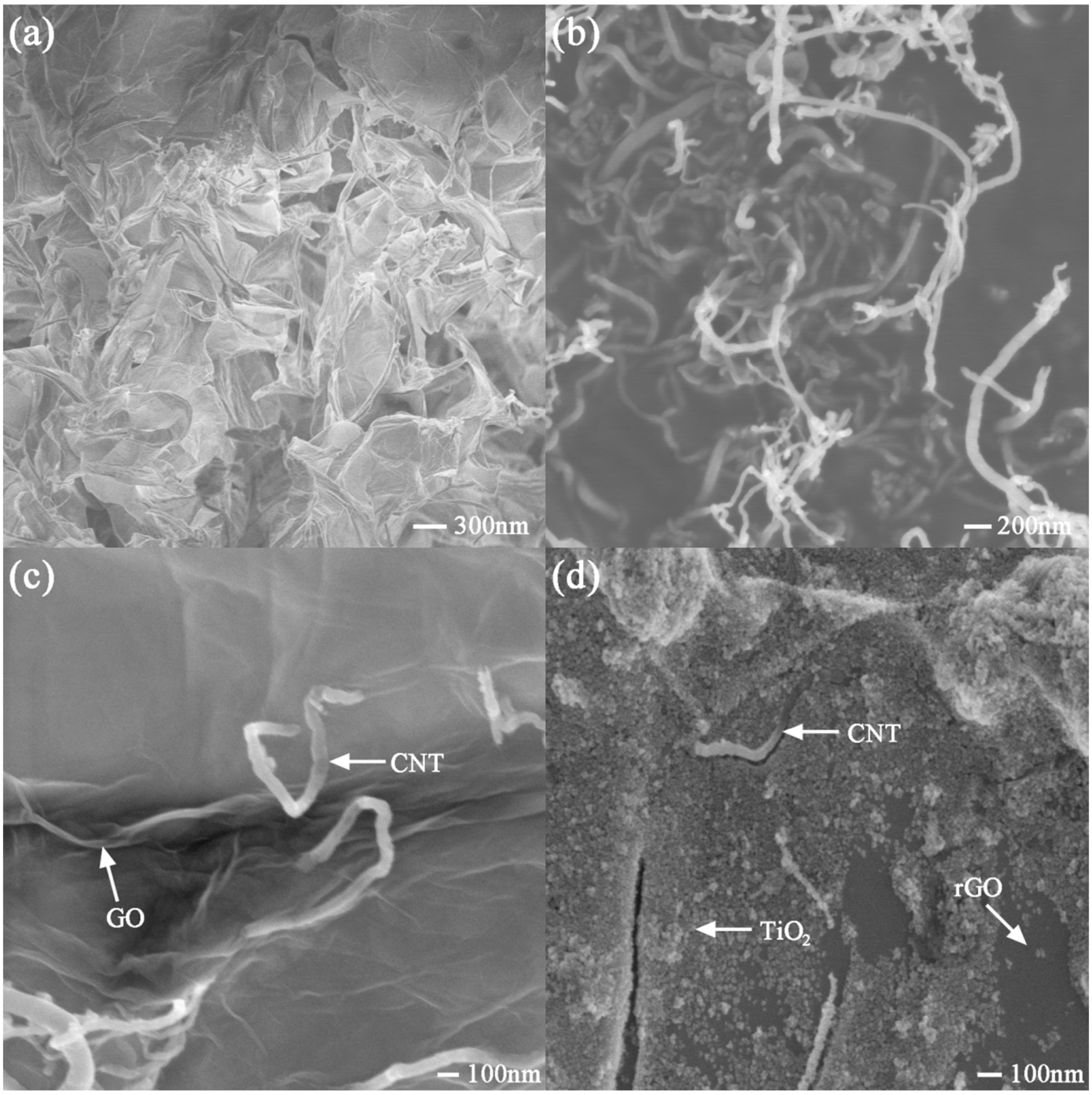

Figure S1. The SEM images of GO, oxide-treated CNTs, CNTs/GO, TiO_2/CNTs/rGO-3%.

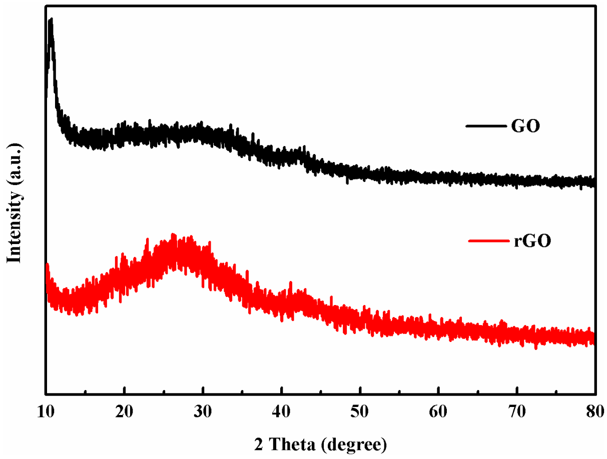

Figure S2. The XRD patterns of rGO and GO.

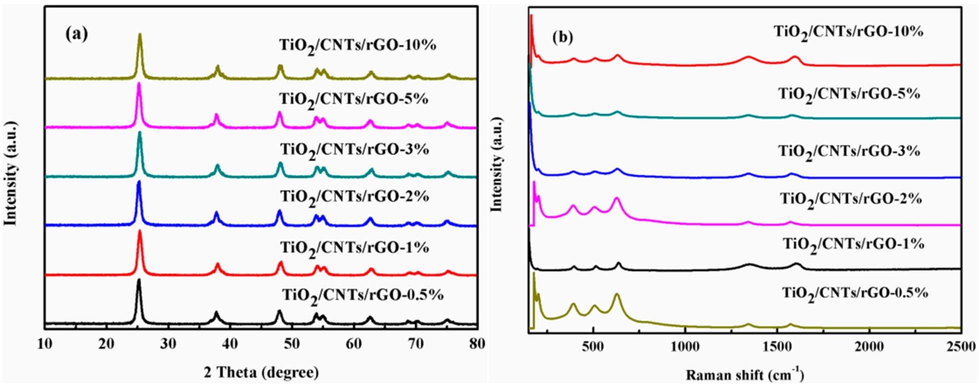

Figure S3. (**a**) The XRD patterns of TiO_2/CNTs/rGO-*x*% (*x* = 0.5, 1, 2, 3, 5, 10); (**b**) The Raman spectra of TiO_2/CNTs/rGO-*x*% (*x* = 0.5, 1, 2, 3, 5, 10).

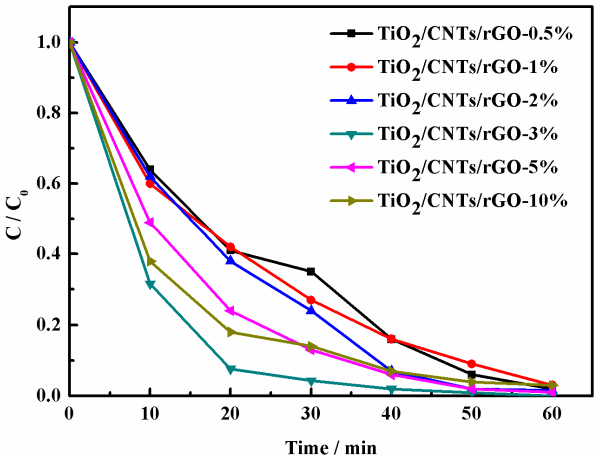

Figure S4. Plot of C/C_0 vs irradiation time of RhB degradation for TiO_2/CNTs/rGO-*x*% (*x* = 0.5, 1, 2, 3, 5, 10).

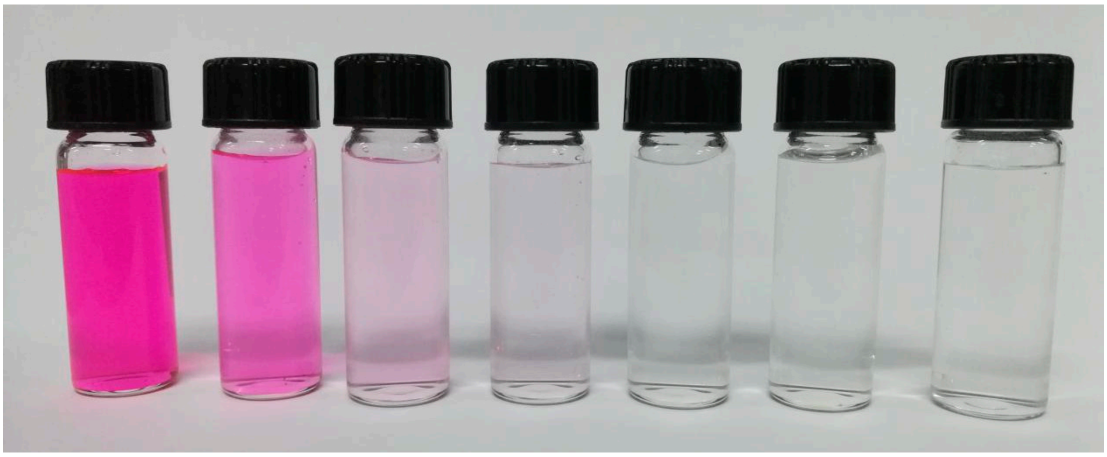

Figure S5. The color change of RhB solution from 0, 10, 20, 30, 40, 50, 60 min under UV-vis light (from left to right) .
